# The Resistance of Soybean Variety Heinong 84 to Apple Latent Spherical Virus Is Controlled by Two Genetic Loci

**DOI:** 10.3390/ijms25042034

**Published:** 2024-02-07

**Authors:** Tingshuai Ma, Ying Zhang, Yong Li, Yu Zhao, Kekely Bruno Attiogbe, Xinyue Fan, Wenqian Fan, Jiaxing Sun, Yalou Luo, Xinwei Yu, Weiqin Ji, Xiaofei Cheng, Xiaoyun Wu

**Affiliations:** 1 College of Plant Protection, Northeast Agricultural University, Harbin 150030, China; s210301066@neau.edu.cn (T.M.); s210301811@neau.edu.cn (Y.Z.); s220301066@neau.edu.cn (Y.Z.); 239803101@neau.edu.cn (K.B.A.); s220302097@neau.edu.cn (X.F.); s231801032@neau.edu.cn (W.F.); a01210397@neau.edu.cn (Y.L.); a01210245@neau.edu.cn (X.Y.); jiweiqinppc@neau.edu.cn (W.J.); 2 College of Life Science, Northeast Agricultural University, Harbin 150030, China; yong@neau.edu.cn

**Keywords:** apple latent spherical virus, bulk segregation analysis, resistance locus, soybean, Heinong 84

## Abstract

Apple latent spherical virus (ALSV) is widely used as a virus-induced gene silencing (VIGS) vector for function genome study. However, the application of ALSV to soybeans is limited by the resistance of many varieties. In this study, the genetic locus linked to the resistance of a resistant soybean variety Heinong 84 was mapped by high-throughput sequencing-based bulk segregation analysis (HTS–BSA) using a hybrid population crossed from Heinong 84 and a susceptible variety, Zhonghuang 13. The results showed that the resistance of Heinong 84 to ALSV is controlled by two genetic loci located on chromosomes 2 and 11, respectively. Cleaved amplified polymorphic sequence (CAPS) markers were developed for identification and genotyping. Inheritance and biochemical analyses suggest that the resistance locus on chromosome 2 plays a dominant dose-dependent role, while the other locus contributes a secondary role in resisting ALSV. The resistance locus on chromosome 2 might encode a protein that can directly inhibit viral proliferation, while the secondary resistance locus on chromosome 11 may encode a host factor required for viral proliferation. Together, these data reveal novel insights on the resistance mechanism of Heinong 84 to ALSV, which will benefit the application of ALSV as a VIGS vector.

## 1. Introduction

Soybean [*Glycine max* (L.) Merrill] is a prominent legume crop for vegetable protein and oil [1]. It provides about 59% of vegetable oil and 70% of plant protein for human and poultry consumption worldwide [2]. To meet the demands of an increasing human population and the quest for a better living standard, it is estimated that global soybean production should at least double by 2050. However, soybean breeding and functional genome studies are significantly hampered by its paleopolyploid genetic background and low transformation efficiency. Virus-induced gene silencing (VIGS) is a widely used RNA-mediated gene knock-down technology [3]. This technology has great significance for crops that are difficult to genetically manipulate, such as soybeans and barley. At present, several soybean-infecting viruses have been engineered into VIGS vectors, including bean pod mottle virus (BPMV), soybean yellow common mosaic virus (SYCMV), cucumber mosaic virus (CMV), tobacco rattle virus (TRV), and apple latent spherical virus (ALSV) [4,5,6,7,8]. However, the application of these VIGS vectors to soybeans is always limited by severe viral symptoms, a narrow infectivity spectrum (they can only infect part of the soybean cultivar), low silencing efficiency, difficult manipulation, etc. For instance, BPMV causes severe viral symptoms in most soybean cultivars, which may significantly affect the observation of the phenotype caused by the silenced gene [5,9]. Understanding the molecular mechanism behind these phenomena can benefit the application of ALSV to soybeans and the pathogenesis of these viruses as well.

ALSV is a typical member of the genus *Cheravirus* in the family *Secoviridae* [10]. Its genome is composed of two positive-sense single-stranded RNA (+ssRNA) molecules that are encapsulated in isometric particles of approximately 25 nm in diameter [11]. Both RNA molecules are polyadenylated at the 3′-end, but their 5′-end is not capped with m7G; instead, it is covalently linked to a viral protein called viral-protein genome-linked protein (VPg). The large +ssRNA (RNA1) encodes one open reading frame (ORF), the product of which is proteolyzed by the viral cysteine proteinase (Pro) into four mature proteins, namely, helicase (Hel), VPg, Pro, and RNA-dependent RNA polymerase (Pol) [11]. The small +ssRNA (RNA2) is also moncistronic, and the translated polyprotein is proteolyzed by Pro into movement protein (MP) and three capsid proteins (Vp25, Vp20, and Vp24) [11]. Although ALSV was isolated from apple [12], it has a wide host range and can infect many plant species in laboratory conditions, such as tobacco, tomato, potato, cucumber, soybean, pea, broad bean, grapevine, cowpea, and red gromwell (*Lithospermum erythrorhizon*) [13,14,15,16,17]. As a latent virus, ALSV usually causes no to very mild symptoms on hosts, can stably survive in host plants for a long period of time, and can be transmitted by the seed and/or pollen of some hosts [18,19]. These advantages make ALSV an ideal VIGS vector for plant genetic research. Indeed, ALSV has been modified into a gene silencing vector for gene function analysis on many crops [13,14,15,16,17]. On soybeans, ALSV causes no or negligible symptoms, has high silencing efficiency, and can invade the embryo [8,20,21]. However, the infectivity and silencing efficiency of ALSV vary with soybean varieties, and many soybean varieties exhibit full resistance to ALSV [8,20,21]. Understanding the resistance mechanism of soybean to ALSV will benefit the application of ALSV for functional genomic studies in soybean. Currently, there is no study on the genetic basis of the infectivity of ALSV on soybeans, and no resistance gene or locus against ALSV has been identified yet. In this study, we report the mapping of the resistance loci and prediction of resistance candidates in Heinong 84, a soybean cultivar from Northeast China, using a hybrid soybean population and high-throughput sequencing-based bulk-segregation analyses (HTS–BSA).

## 2. Results

### 2.1. Screening of Susceptible Soybean Varieties in Northeast China

**Figure 1:**
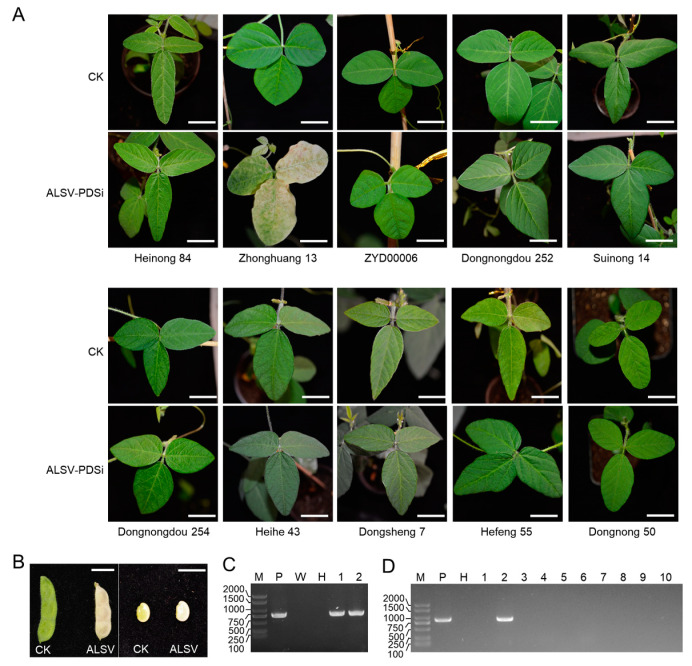
Phenotypes and RT–PCR analyses of ALSV-PDSi infectivity on different soybean varieties. (**A**) Symptoms of the systemic leaves of 10 soybean varieties inoculated by ALSV-PDSi at 20 dpi. Scale bars, 5 cm. (**B**) Phenotypes of bean pods (**left**) and seeds (**right**) produced by seedlings of Zhonghuang 13 inoculated by ALSV-PDSi (ALSV) or buffer (CK). Scale bars, 1 cm. (**C**) RT–PCR detection of ALSV genome in the seed coat and seed. Lanes M, P, W, and H indicate the DNA ladder, plasmid control, mock control, and healthy soybean seed control, respectively. Lanes 1 and 2 are seed coat and embryo showing photobleaching phenotype, respectively. (**D**) RT–PCR analysis of the presence of viral genome in the systemic leaves of the ten soybean cultivars. Lanes 1 to 10 are Heinong 84, Zhonghuang 13, ZYD00006, Dongnongdou 252, Suinong 14, Dongnongdou 254, Heihe 43, Dongsheng 7, Hefeng 55, and Dongnong 50, respectively.

The Northeast area is the most significant soybean-producing region in China. To explore the soybean cultivars suitable for VIGS application in this area, we mechanically inoculated the first true leaf of twelve-day-old seedlings of ten major soybean varieties from the Northeast region of China by ALSV-PDSi, an ALSV infectious clone harboring a fragment of the soybean *phytoene desaturase* (*PDS*) gene between MP and VP25 for tracking virus infection [20]. Zhonghuang 13, which has been confirmed to be susceptible to ALSV, was included as a positive control [20]. As expected, a photobleaching symptom was observed on the upper uninoculated leaves of Zhonghuang 13 at 20 days post-inoculation (dpi) (Figure 1A). Moreover, the seed coat and seeds of Zhonghuang 13 also showed a photobleaching symptom (Figure 1B), suggesting that ALSV may be seed-transmittible in Zhonghuang 13. However, no visible photobleaching symptom was observed on the upper uninoculated leaves of the ten soybean varieties throughout their growth period (Figure 1A). The presence of ALSV-PDSi on the upper uninoculated leaves was confirmed by a reverse transcription-polymerase chain reaction (RT–PCR). The results showed that ALSV RNA was detected in the seed coat and embryo of Zhonghuang 13, but not in any of these soybean varieties (Figure 1C,D), which further confirm that only a portion of soybean varieties are susceptible to ALSV.

### 2.2. Genetic Analysis of the Resistance of Heinong 84 to ALSV

Previously, we constructed a soybean hybrid population by crossing Heinong 84 and Zhonghuang 13 to locate the resistance gene against the soybean mosaic virus strain N3 in Heinong 84 [22]. We thus decided to take advantage of this hybrid population to analyze the genetic basis of the trait of ALSV resistance of Heinong 84. A total of 100 F2-generation seedlings were mechanically inoculated by ALSV-PDSi. The infection of ALSV-PDSi on each plant was confirmed by both the photobleaching phenotype and RT–PCR. The results showed that 40 out of the 100 F2-generation seedlings displayed a photobleaching phenotype and the presence of viral genomic RNA on the upper systemic leaves. The remaining 60 F2-generation seedlings showed no photobleaching phenotype throughout the growth period and were absent of viral genomic RNA on the upper systemic leaves (Table 1). The segregation ratio did not match 3:1 (χ^2^ = 4.4672; *p* = 0.03445), but matched 9:7 (χ^2^ = 0.185; *p* = 0.6673; Table 1), indicating that this resistance trait of Heinong 84 may be controlled by two genes.

### 2.3. Location of the Resistance Loci in Heinong 84 by HGS–BSA

**Figure 2:**
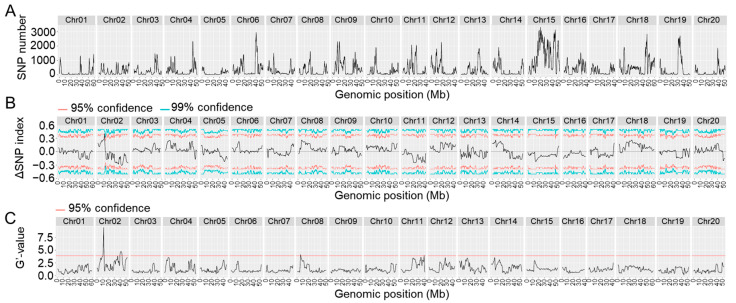
Distribution and visualization of SNPs. (**A**) Distribution of all SNPs on the twenty soybean chromosomes. (**B**) The ΔSNP index patterns of NGS-based BSA. (**C**) Pattern of the G′-value. The X-axis shows chromosome positions for all twenty chromosomes, while the Y-axis represents the count of SNPs (**A**), ΔSNP index (**B**), or G′-value (**C**). The ΔSNP index was computed as 1 Mb interval with a 10 kb stepwise. The red and cyan lines in panels (**B**,**C**) represent the expected values at 95% confidence (*p* < 0.05) and 99% confidence (*p* < 0.01), respectively.

We inoculated the F3-generation of the hybrid population and used the same standard to select resistant and susceptible offsprings. A total number of 30 susceptible individuals (showing a photobleaching phenotype) and 30 resistant individuals were selected for further analyses. Due to inoculation efficiency, the resistant pool may include a very small number of susceptible individuals. Equal amounts of the genomic DNA of each resistant or susceptible individual were pooled as the resistant or susceptible pool, respectively. The two pools and the genomic DNA of Heinong 84 were then sequenced by HTS. After adaptor trimming and discarding low-quality reads, we obtained 29.8, 30.7, and 27.3 billion high-quality reads (quality score ≥ 30) of the resistant pool, susceptible pool, and Heinong 84, respectively. These data were mapped to the reference genome of Zhonghuang 13, and single-nucleotide polymorphism (SNP) sites were thereby retrieved. A total number of 366,633 SNPs distributed on the 20 chromosomes of Zhonghuang 13 were found. Subsequent BSA revealed two genomic intervals, one 4.62 Mb genomic interval on chromosome 2 (39,213,950–43,829,476 bp; *p* = 0.00004) with 99% confidence and another 6.41 Mb genomic interval on chromosome 11 (19,104,714–25,514,536 bp; *p* = 0.00040), were identified as the potential quantitative trait loci (QTLs) (Figure 2; Table 2). Taken together, these data confirm that the resistance of Heinong 84 to ALSV is controlled by two genes, hereinafter referred to as *resistance to ALSV locus 1* (*R_ALSV_-L1*) and *R_ALSV_-L2*, respectively.

### 2.4. Dissection the Genetic Basis of RALSV-L1 and RALSV-L2 Using CAPS Markers

The CAPS-marker-based test is a rapid, reliable, and co-dominant method that has been successfully applied for QTL mapping, molecular-marker-assisted breeding, and inheritance analysis [23,24]. We thus designed three pairs of CAPS primers per locus for further inheritance analysis. PCR using the total DNA extracted from the parents followed by restriction enzyme digestion showed that the primers based on SNP3999 and SNP2115 resulted in partial digestion (Figure S1), and thus were excluded in the subsequent experiments. All individuals in the susceptible and resistance pools were then analyzed by the four pairs of CAPS primers. The results showed that the primers based on SNP4209 on chromosome 2 (C2-SNP4209-F and C2-SNP4209-R) displayed a higher cosegregation confidence than that based on SNP4232 (Figures S2 and S3), while the two pairs of primers on chromosome 11 had similar performance (Figures S4 and S5). We then determined the genotype of each plant in the susceptible and resistance pools based on the results of the CAPS assay. For statistical purposes, the ALSV-associated loci on chromosome 2 in Heinong 84 and Zhonghuang 13 were defined as *A1* and *a1*, respectively. Partially digested samples were recognized as heterozygous. The results showed that most individuals that were homologous to *A1* or *a1* were resistant and susceptible to ALSV, respectively (Table 3), indicating that *A1* is a dominant resistance gene. Interestingly, half of heterozygous individuals are resistant and half are susceptible to ALSV (Table 3), suggesting that *A1* may be a dose-dependent dominant resistance gene and/or that the genotype of the other locus together determines the phenotype when this locus is heterozygous.

Similar genotype analyses were performed for the resistance locus on chromosome 11, which was defined as *B2* and *b2* in Heinong 84 and Zhonghuang 13, respectively. The results showed that the majority of individuals that were homologous to *B2* or *b2* were susceptible and resistant to ALSV, respectively (Table 3), indicating that the ALSV-associated locus on chromosome 11 may encode a recessive resistance gene, and this gene, even if it is homozygous, can only provide partial resistance. Interestingly, only one heterozygous plant was identified in this study (Table 3); thus, it could not be further analyzed.

We further performed a correlation analysis of the two loci (Table 3). The Fisher’s exact test confirmed that the two ALSV-associated loci were significantly associated with the resistance phenotype (*p* = 0.003263). As expected, almost all individuals with the genotypes *A1A1b2b2* and *A1a1b2b2* are resistant (Table 3), further confirming that *A1* is a dominant resistance gene, while *b2* is a recessive resistance gene. The majority of individuals with the genotypes *a1a1B2b2*, *a1a1b2b2*, and *a1a1B2B2* are also susceptible (Table 3), suggesting that *b2* cannot provide full resistance without the *A1* gene. Interestingly, about half of the individuals with the genotypes of *A1A1B2B2* or *A1a1B2B2* are resistant and half are susceptible to ALSV (Table 3), confirming that *A1* is a dominant gene that may function by directly inhibiting viral proliferation, while *B2* may encode a host factor that is required for viral proliferation.

### 2.5. Prediction of Candidate Genes in the Resistance Loci RALSV-L1 and RALSV-L1

Using the reference genome of Zhonghuang 13, a total of 266 protein-coding, microRNA (miRNA), and transfer RNA (tRNA) genes were identified in the resistance interval on chromosome 2, among which 235 genes exhibited variations between Heinong 84 and Zhonghuang 13. Considering the effects of the SNP on the gene where it is located, these genes were divided into three groups: low-potential candidates, moderate-potential candidates, and high-potential candidates. The low-potential candidates had SNPs that caused synonymous mutations; the moderate-potential candidates had SNPs that caused missense mutations and variation in the untranslated region (UTR) and/or intron; while the high-potential candidates contained SNPs that caused splice acceptor variants and intron variants, stop codon loss, frameshift variants, and stop codon gain. Based on this criterion, a total of 69 and 10 moderate- and high-potential candidates were identified in the resistance locus, respectively (Table S1). The same analyses were performed on the resistance locus on chromosome 11. A total of 238 protein-coding, miRNA, and tRNA genes were identified in the resistance interval on chromosome 11, among which 191 and 29 were moderate- and high-potential candidates (Table S2). A Gene Ontology (GO) and Kyoto Encyclopedia of Genes and Genomes (KEGG) search showed that the candidates of *R_ALSV_-L1* and *R_ALSV_-L2* belonged to various pathways and had varied biological functions. Nevertheless, we noticed that several candidates of *R_ALSV_-L1* and *R_ALSV_-L2* belonged to the same protein complex or biological pathways, namely, the cleavage and polyadenylation specificity factor (CPSF) complex, leucine-rich repeat-containing protein, protein ubiquitination, and microtubule motors (Tables S1 and S2).

### 2.6. The Resistance Is Not Associated with Innate Immunity

**Figure 3:**
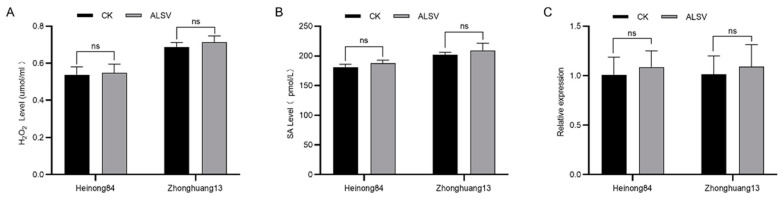
The resistance of Heinong 84 to ALSV is not associated with innate immunity. (**A**) Bar chart showing the accumulation of H_2_O_2_ in soybean plants inoculated by ALSV or buffer at 48 hpi. (**B**) Bar chart showing the accumulation of SA in soybean plants inoculated by ALSV or buffer at 48 hpi. (**C**) Bar chart showing the transcript levels of *PR1* in soybean plants inoculated by ALSV or buffer at 48 hpi. The *GmCons6* gene (Glyma.12G051100) was used as the internal control. ns indicates *p* > 0.05 in the Student’s *t*-test. Experiments were replicated three times with consistent results.

To test whether the dominant locus *R_ALSV_-L1* in Heinong 84 was associated with innate immunity, we compared the content of hydrogen peroxide (H_2_O_2_), a marker molecule of biotic stresses in plants. Thus, we inoculated seedlings of Heinong 84 and Zhonghuang 13 with ALSV or buffer and determined the content of H_2_O_2_ in the inoculated leaf tissue was at 48 h post-inoculation (hpi). The results showed that there was no significant difference in the contents of H_2_O_2_ in the leaves of Heinong 84 and Zhonghuang 13 inoculated by ALSV or buffer at 48 hpi (Figure 3A), indicating that the inoculation of ALSV does not induce the accumulation of H_2_O_2_ in Heinong 84 and Zhonghuang 13. We also compared the content of salicylic acid (SA), the key phytohormone of biotic stresses in plants, with the content of ALSV infection. The results showed that the SA content in Heinong 84 and Zhonghuang 13 was not significantly upregulated after the inoculation of ALSV at 48 hpi (Figure 3B). Finally, we directly compared the expression of *pathogenesis-related genes 1* (*PR1*), the marker gene of plant innate immunity, in Heinong 84 and Zhonghuang 13. Quantitative PCR (qPCR) results showed that there was no significant difference in the expression of *PR1* in the leaves of Heinong 84 and Zhonghuang 13 inoculated by ALSV or buffer at 48 hpi (Figure 3C). Together, we concluded that the resistance in Heinong 84 to ALSV is not controlled by innate immunity but instead may be controlled by non-immune-related mechanisms, e.g., RNA silencing, translation repression, essential host factors for virus proliferation, or atypical dominant viral resistance protein [25].

### 2.7. There Are Resistance Genes Other Than R_ALSV_-L1 or R_ALSV_-L2 in Soybeans

**Figure 4:**
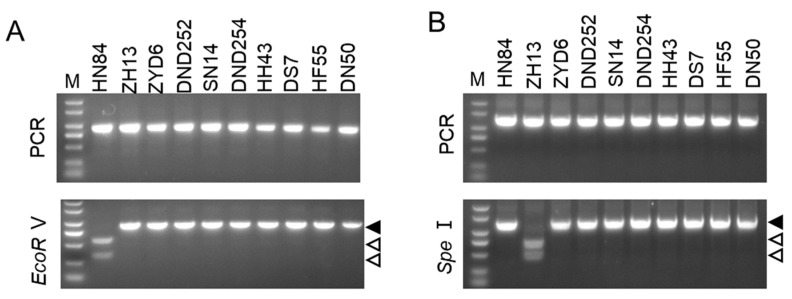
CAPS results of the eight soybean cultivars. (**A**,**B**) Gel electrophoresis results of the PCR products of primer pairs based on SNP4209 (**A**) and -SNP2130 (**B**) before and after restriction enzyme digestion. Lanes 1 to 10 represent Heinong 84 (HN84), Zhonghuang 13 (ZH13), ZYD00006 (ZYD6), Dongnongdou 252 (DND252), Suinong 14 (SN14), Dongnongdou 254 (DND254), Heihe 43 (HH43), Dongsheng 7 (DS7), Hefeng 55 (HF55), and Dongnong 50 (DN50), respectively. The PCR amplicons are indicated by solid arrowheads and digested fragments are indicated by hollow arrowheads.

We further analyzed the resistance of the other eight soybean varieties, namely, Heihe 43, Dongsheng 7, Hefeng 55, ZYD00006, Dongnongdou 252, Suinong 14, Dongnongdou 254, and Dongnong 50, using the two pairs of CAPS primers based on SNP4209 and SNP2130. The results showed that the ALSV-resistance-associated locus on chromosome 2 of all eight soybean cultivars had the same genotype as Zhonghuang 13, while the ALSV-resistance-associated locus on chromosome 11 of these soybean cultivars was the same as Heinong 84 (Figure 4). These data suggest that the resistance of these cultivars may be controlled by additional resistance genes, and these primers are not suitable for dissecting the resistance of these soybean cultivars to ALSV.

## 3. Discussion

Despite the advantages of ALSV as a VIGS vector for soybean genome study and some soybean cultivars, such as Wyandot, Magellan, Jack, Qihuang 34, Andou 203, Nannong 1138-2, Nannong 47, Zhonghuang 13, Shanning 29, and Xiangdou 4, having been identified as susceptible cultivars [8,20,21], many soybean cultivars display complete resistance to ALSV. We also found that all ten major soybean cultivars from the Northeast region of China were completely resistant to ALSV. Understanding the genetic mechanisms underlying ALSV resistance is of great significance for the application of ALSV to soybeans. However, no resistance-associated genetic locus or DNA marker has been characterized in soybeans. Based on simple genetic background comparisons, it was speculated that the resistance was determined by one or several genes [21]. In this study, the resistance of Heinong 84 to ALSV was analyzed in detail by a hybrid population crossed from the resistant cultivar Heinong 84 and the susceptible cultivar Zhonghuang 13. Our BSA and CAPS assay data clearly showed that the resistance of Heinong 84 is associated with two genetic loci located on chromosomes 2 and 11, respectively. These data allow us to understand, for the first time, the genetic basis of soybean resistance to ALSV. Interestingly, the results of the CAPS assay of the other eight soybean varieties using the same primers reveal that there are other resistance loci in soybean. Thus, the resistance of soybean to ALSV is more complex than previously thought.

CAPS primers were also designed for the rapid identification of resistance loci and for dissecting the genetic basis of the resistance. The data from the CAPS assays further confirmed that the resistance of ALSV is controlled by two loci: one dominant locus on chromosome 2 and another recessive locus on chromosome 11. Interestingly, a detailed dissection of the relationship between the genotype and resistance phenotype suggests that the two loci have very complex genetic relationships: the locus on chromosome 2 plays a dominant but dose-dependent role in resisting ALSV, while the locus on chromosome 11 only has an auxiliary role. Based on these observations, it is possible that *R_ALSV_-L1* may encode atypical dominant viral resistance protein (ADVRP), and *R_ALSV_-L2* belongs to a key host factor required for ALSV proliferation. Indeed, the inoculation of ALSV did not induce the accumulation of H_2_O_2_ or SA and did not stimulate the expression of *PR1*. At present, several ADVRPs have been characterized, such as the restricted TEV movement 1 (RTM1) that confers resistance to several potyviruses [26], the jacalin-type lectin required for potexvirus resistance 1 (JAX1) that confers broad-spectrum resistance to potexviruses [27], the tomato Tm-1, which confers resistance to tomato mosaic virus (ToMV, a tobamovirus) [28,29], and h-type thioredoxin (ZmTrxh), which provides maize with sugarcane mosaic virus (SCMV, a potyvirus) resistance [30]. However, no candidate was found to be homologous to these ADVRPs. Thus, *R_ALSV_-L1* may encode a novel ADVRP. Viruses are obligate intracellular parasites and require many host factors to accomplish their infection cycle, e.g., protein expression, genome replication, and intercellular movement. The incompatible or unfavorable interaction between viral proteins or genomes will cause a delay or even complete failure of the infection [31]. The eukaryotic initiation factor 4E (eIF4E) and eIFiso4E are the two most documented recessive resistance genes [32]. However, we did not find either eIF4E or eIFiso4E in the candidate gene list. No candidate was found to be homologous to other antiviral recessive resistance genes, such as essential for potexvirus accumulation 1 (EXA1) and the translationally controlled tumor protein TCTP [33,34]. Thus, further investigations are needed to fully illustrate the function of the recessive gene in the *R_ALSV_-L2* locus.

## 4. Materials and Methods

### 4.1. Soybean Varieties and Growth Conditions

Soybean varieties Heihe 43, Dongsheng 7, Hefeng 55, ZYD00006, Dongnongdou 252, Suinong 14, Dongnongdou 254, Heinong 84, Zhonghuang 13, and Dongnong 50 were grown in pot in a growth chamber with 50% humidity and 16:8 (light: dark) photoperiod at 26 °C. The hybrid population from the crossing between Heinong 84 and Zhonghuang 13 has been reported previously [22].

### 4.2. ALSV Inoculation

The ALSV infectious clone on the pCB301 backbone has been reported previously [20]. Soybeans were sap-inoculated as described earlier with a few modifications [20]. In brief, the two plasmids pALSV-R1 and pALSVR2-PDSi harboring RNA1 and RNA2 of ALSV, respectively, were transformed into the *Agrobacterium tumefaciens* strain GV3101 (plus pSoup-p19) by electroporation. An equal amount of the bacteria harboring pALSV-R1 and pALSVR2-PDSi were mixed and infiltrated into *Nicotiana benthamiana* leaves. At 20 dpi, the systemic leaves were harvested for viral particle enrichment as described [20]. The virion solution was used for the subsequent sap inoculation of the first true leaf of soybean seedlings that had been pre-dusted with 600 mesh carborundum powder. After inoculation, the leaf was rinsed with distilled water and covered with a prewetted paper towel to prevent dehydration. The seedlings were then put back into the growth chamber with normal care measures.

### 4.3. RNA Extraction and RT–qPCR

Total RNA was extracted from soybean leaves using the Eastep^®^ Super Total RNA Extraction Kit [Promega, Beijing, China] following the supplied instructions. First-strand complementary DNA (cDNA) was synthesized using the HiScript III 1st Strand cDNA Synthesis (+gDNA wiper) Kit (Vazyme Biotech, Nanjing, China) with random hexamers and Oligo-dT_20_. qPCR was conducted in a 20 μL reaction volume, comprising 1 μL of 200 ng/μL cDNA, 0.4 μL each of 10 mmol/μL forward and reverse primers, 10 μL of 2 × ChamQ Universal SYBR qPCR Master Mix (Vazyme Biotech), and 8.2 μL sterilized ultrapure water. All primers used in the present study are listed in Table 4.

### 4.4. DNA Extraction, DNA Pool Preparation, and HTS

Total genome DNA was isolated using the FastPure Plant DNA Isolation Mini Kit (Vazyme Biotech). DNA pools were prepared as described earlier with few modifications [22]. The susceptible and resistant pools included 30 susceptible and resistant soybean DNA of 6 μg DNA per sample, respectively. HTS was performed by the Illumina HiSeqTM 2500 platform in Hangzhou Lianchuan Biotechnology Co., Ltd. (Hangzhou, Zhejiang, China).

### 4.5. Bulk Segregation Analysis (BSA)

The adapter sequence on reads from the HiSeqTM 2500 platform was trimmed and low-quality reads were discarded using a Trimmomatic v0.39 [35]. The resulting high-quality reads were mapped to the reference genome of Zhonghuang 13 (CNCB accession GWHAAEV00000000.1) using HiSat2 v2.2.1 with the end-to-end model [36]. SNP was called using the samtools v1.15 after removing duplication [37]. QTLseqr (v0.7.5.2) was used to localize resistance-associated locus (SNPs with a sample depth below 10 or total depth below 30 were removed) [38].

### 4.6. Design and Validation of CAPS Primers

The mapping results from HiSat2 were utilized to identify SNPs inside the resistance locus using bedtools v2.30 [39]. The SNP2CAPS v0.6 software was employed to transform the SNPs into CAPS markers [23]. CAPS primers were designed using SnapGene 4.1.9 with the default parameters. PCR was carried out in a 20 μL volume system in a T30D tri-block super-gradient PCR system (LongGene, Hangzhou, China). The thermal cycle contains a pre-denaturation step at 95 °C for 3 min, 30 cycles of denaturation at 95 °C for 30 s, annealing at the primer melting temperature (Tm) for 30 s, extension at 72 °C for 15 s, and a final extension step at 72 °C for 5 min. Restriction enzyme digestion was performed in a 20 µL system, comprising 2.0 µL of 10× CutSmart buffer, 0.5 µL of restriction enzyme, e.g., *Hind* III, *EcoR* V, and *Nhe* I (New England Biolabs, Beijing, China), 10 µL of PCR product, and 7.5 µL of ddH_2_O. The restriction enzyme digestion mixture was incubated at 37 °C for 30 min, and then treated at 85 °C for 5 s to deactivate the enzyme. Subsequently, the mixtures of PCR and restriction enzyme digestion were analyzed by 1% agarose gel electrophoresis. ImageJ (https://imagej.net/software/imagej/) was used to analyze the amplified bands, and statistical analyses were performed using Fisher’s Exact test.

### 4.7. SA and H_2_O_2_ Quantification

The Plant Salicylic Acid (SA) ELISA Kit (Spbio, Wuhan, China) was used to determine the concentration of SA according to the manufactural instructions. The BioTech Epoch Full Wavelength Enzyme Labeler (Agilent, Beijing, China) was used to read the optical density at a wavelength of 450 nm (OD450). Every sample was technically triple-replicated, and the OD450 read was corrected by the value of the blank control, and then compared to the OD450 value of the healthy control (leaf tissue inoculated with buffer).

H_2_O_2_ content was measured using the Hydrogen Peroxide Assay Kit (Boxbio Biotech, Beijing, China] following the supplied manual. In brief, about 0.1 g of leaf tissue was homogenized in 1 mL of solution I; the resulting homogenate was clarified by centrifugation at 8000× *g* for 30 min at 4 °C. The supernatant was transferred to a new test tube, and 100 µL of solution II, 200 µL of solution III, and 1 mL of solution IV were sequentially added. The solution was then mixed well through a vortex. After incubation at room temperature for 5 min, 1 mL of the reaction solution was transferred to a measuring cuvette, and the absorbance at 415 nm was read by a Thermo Fisher UV-Vis spectrophotometer (ThermoFisher Scientific, Shanghai, China). Finally, the value at OD415 was plotted against the standard curve to calculate the concentration of H_2_O_2_.

## 5. Conclusions

In conclusion, the resistance of Heinong 84 to ALSV is associated with two genetic loci on chromosomes 2 and 11, respectively. The CAPS assay further implies that the resistance is possibly controlled by the complex interaction between an ADVRP and the host factor required for ALSV proliferation.

## Figures and Tables

**Table 1:** Genetic analysis of the resistance of the F2-generation hybrid populations to ALSV.

Parent and Offspring	Resistant	Susceptible	Total	Theoretical Separation Ratio	χ^2^	*p*
Heinong 84	6	0	6			
Zhonghuang 13	0	6	6			
F_2_	60	40		3:1	4.4672	0.03445
				9:7	0.185	0.6673

**Table 2:** Characterization of the resistance loci in Heinong 84.

Chromosome	Start Position	End Position	Length	Number of SNPs	*p* Value
2	39,213,950	43,829,476	4,615,526	1710	0.00004
11	19,104,714	25,514,536	6,409,822	5666	0.00040

**Table 3:** Statistics and correlation analysis of genotype and phenotype of F3 population.

Genotype	Resistant	Susceptible	Total	*p* Value
*A1A1*	12	3	15	
*A1a1*	13	9	22	
*a1a1*	5	18	23	
*A1A1* + *A1a1* + *a1a1*	30	30	60	0.001115
*B2B2*	11	19	30	
*B2b2*	0	1	1	
*b2b2*	19	10	29	
*B2B2* + *B2b2* + *b2b2*	30	30	60	0.03789
*A1A1b2b2*	10	1	11	
*A1a1b2b2*	8	3	11	
*A1A1B2B2*	2	2	4	
*A1a1B2B2*	5	6	11	
*a1a1B2B2*	4	11	15	
*a1a1b2b2*	1	6	7	
*a1a1B2b2*	0	1	1	
All	30	30	60	0.003263

**Table 4:** Primers used in the research.

Primer Names	Sequences (5′-3′)	Usage
GmPR1-F	TGCTAATCAACGCAAAGG	RT–qPCR
GmPR1-R	CATCCAAGACGCACCGAG	RT–qPCR
Cons6-F	AAGTTAGGAGCCCAAGACAT	RT–qPCR
Cons6-R	AGCGAGTTCATTGAAGCAGA	RT–qPCR
C2-SNP3999-F	GGACATACTTGCAAGTTTAGGG	CAPS analysis
C2-SNP3999-R	GAGGTTGGCCTATAAACCTC	CAPS analysis
C2-SNP4209-F	GCAGGGCATAAACTTTCAATC	CAPS analysis
C2-SNP4209-R	CAGTTAAGTGATTGCATTGGC	CAPS analysis
C2-SNP4232-F	CCACATCACTAACCTAAAGGC	CAPS analysis
C2-SNP4232-R	TTCACTTATGCCCAAGGGC	CAPS analysis
C11-SNP2115-F	GATCGAGGTAGTGGTAGACATC	CAPS analysis
C11-SNP2115-R	CACTTGTGCAGCAGAATCGTG	CAPS analysis
C11-SNP2130-F	GCAGCGGTTCAAAACCGTCC	CAPS analysis
C11-SNP2130-R	TGATCCTGAAGGTTGAGGATGC	CAPS analysis
C11-SNP2298-F	ACTCTGAAGCGTATCCATGACC	CAPS analysis
C11-SNP2298-R	TGTTGACGGTTTATTTAGATGAC	CAPS analysis
ALSV2-F	GCTCGTCACCTGTTCAGCTC	RT–PCR
ALSV2-R	CTAGGTGTAACCAGCTTTGAGC	RT–PCR
MP944-F	CTGATGGTGTCCTCAAAGAGG	RT–PCR
VP195-R	GGTAAATTCTGGAGTAGAAG	RT–PCR

## Data Availability

All data are available within the Article and Supplementary Files. All constructs are available upon request.

## Supplementary Materials

The following supporting information can be downloaded at: https://www.mdpi.com/article/10.3390/ijms25042034/s1.
