# Supplementary material for: The Resistance of Soybean Variety Heinong 84 to Apple Latent Spherical Virus Is Controlled by Two Genetic Loci
Source: Int J Mol Sci. 2024 Feb 7;25(4):2034. doi: 10.3390/ijms25042034 (PMC10889123; doi:10.3390/ijms25042034)
Supplement: Supplementary file 1 [file ijms-25-02034-s001.zip › Supplimentary Figures.pptx]

## Slide 1
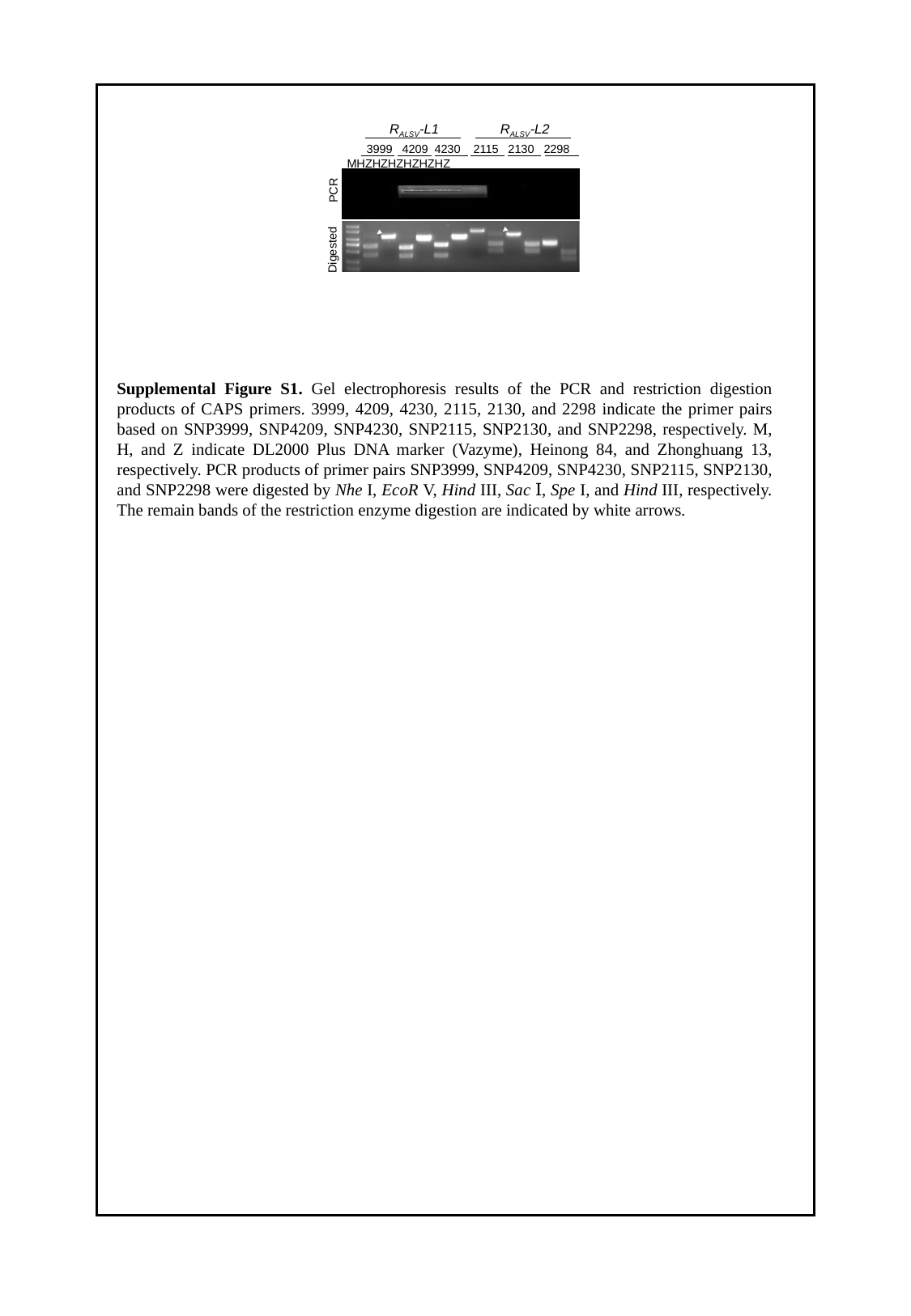

RALSV-L1
RALSV-L2
 3999 4209 4230 2115 2130 2298
MHZHZHZHZHZHZ
PCR
Digested
Supplemental Figure S1. Gel electrophoresis results of the PCR and restriction digestion products of CAPS primers. 3999, 4209, 4230, 2115, 2130, and 2298 indicate the primer pairs based on SNP3999, SNP4209, SNP4230, SNP2115, SNP2130, and SNP2298, respectively. M, H, and Z indicate DL2000 Plus DNA marker (Vazyme), Heinong 84, and Zhonghuang 13, respectively. PCR products of primer pairs SNP3999, SNP4209, SNP4230, SNP2115, SNP2130, and SNP2298 were digested by Nhe I, EcoR V, Hind III, Sac Ⅰ, Spe I, and Hind III, respectively. The remain bands of the restriction enzyme digestion are indicated by white arrows.

## Slide 2
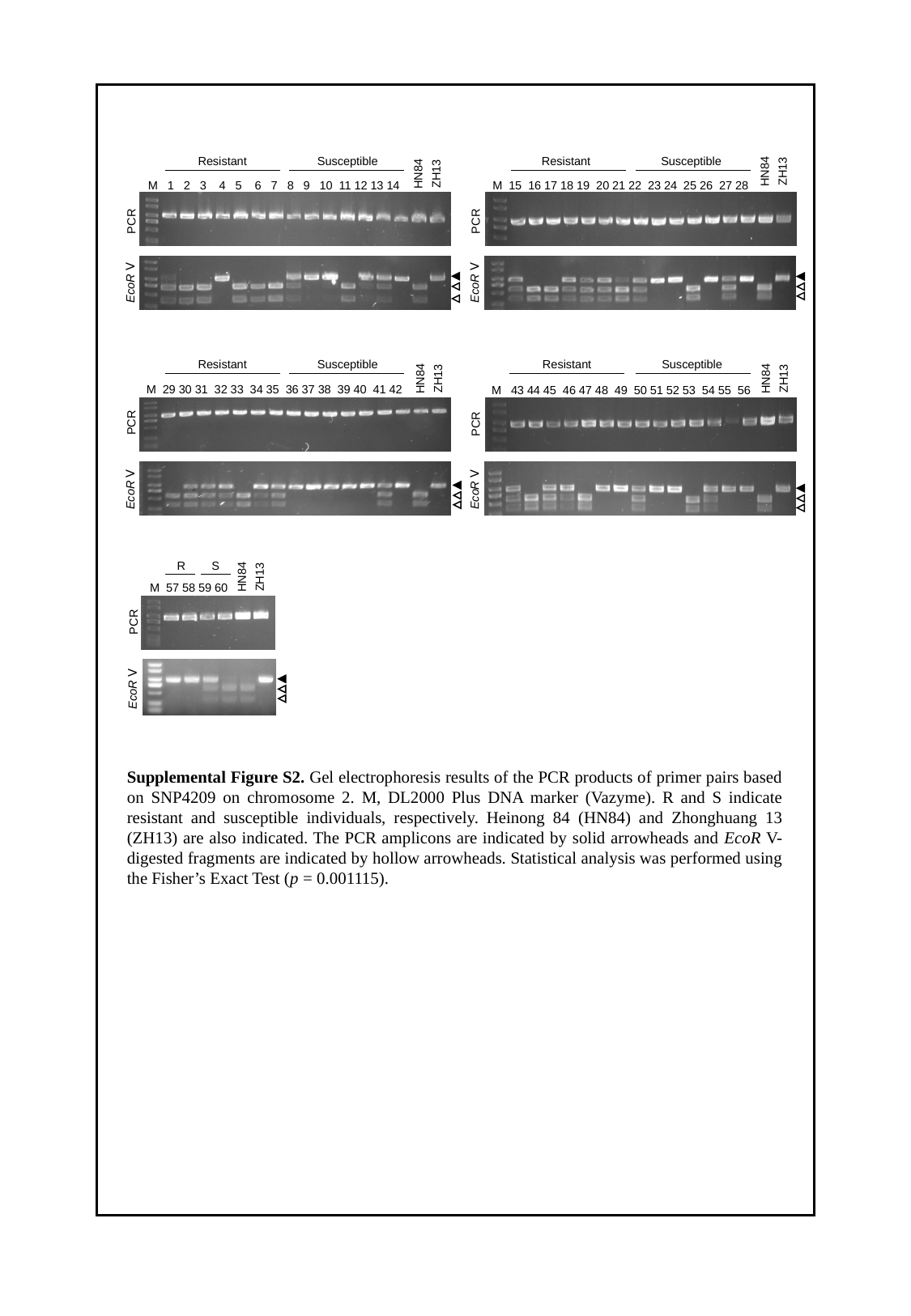

Resistant
Susceptible
Resistant
Susceptible
ZH13
HN84
ZH13
HN84
M 1 2 3 4 5 6 7 8 9 10 11 12 13 14
M 15 16 17 18 19 20 21 22 23 24 25 26 27 28
PCR
PCR
EcoR Ⅴ
EcoR Ⅴ
Resistant
Susceptible
Resistant
Susceptible
ZH13
ZH13
HN84
HN84
M 29 30 31 32 33 34 35 36 37 38 39 40 41 42
M 43 44 45 46 47 48 49 50 51 52 53 54 55 56
PCR
PCR
EcoR Ⅴ
EcoR Ⅴ
R
S
ZH13
HN84
M 57 58 59 60
PCR
EcoR Ⅴ
Supplemental Figure S2. Gel electrophoresis results of the PCR products of primer pairs based on SNP4209 on chromosome 2. M, DL2000 Plus DNA marker (Vazyme). R and S indicate resistant and susceptible individuals, respectively. Heinong 84 (HN84) and Zhonghuang 13 (ZH13) are also indicated. The PCR amplicons are indicated by solid arrowheads and EcoR V-digested fragments are indicated by hollow arrowheads. Statistical analysis was performed using the Fisher’s Exact Test (p = 0.001115).

## Slide 3
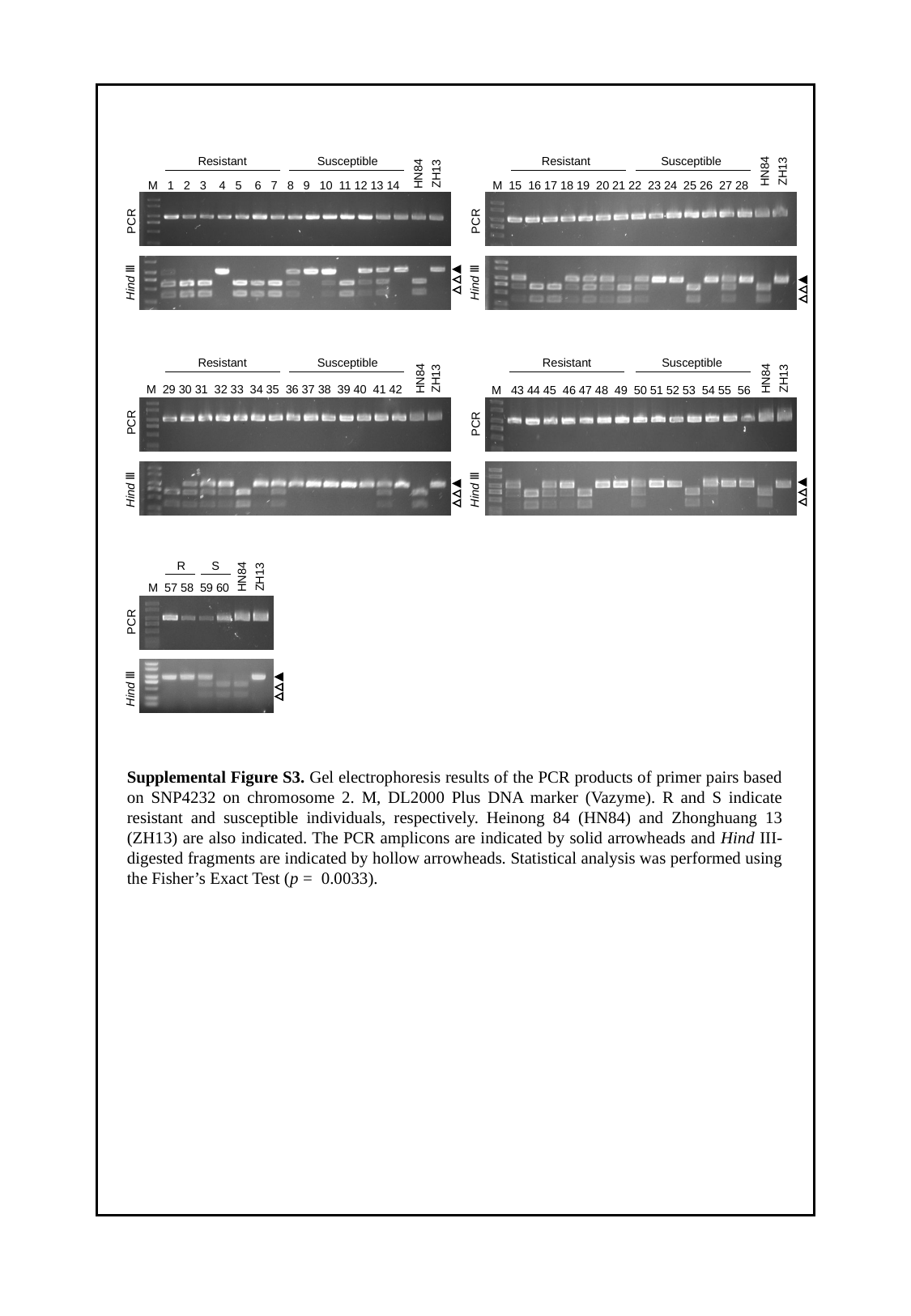

Resistant
Susceptible
Resistant
Susceptible
ZH13
HN84
ZH13
HN84
M 1 2 3 4 5 6 7 8 9 10 11 12 13 14
M 15 16 17 18 19 20 21 22 23 24 25 26 27 28
PCR
PCR
Hind Ⅲ
Hind Ⅲ
Resistant
Susceptible
Resistant
Susceptible
ZH13
ZH13
HN84
HN84
M 29 30 31 32 33 34 35 36 37 38 39 40 41 42
M 43 44 45 46 47 48 49 50 51 52 53 54 55 56
PCR
PCR
Hind Ⅲ
Hind Ⅲ
R
S
ZH13
HN84
M 57 58 59 60
PCR
Hind Ⅲ
Supplemental Figure S3. Gel electrophoresis results of the PCR products of primer pairs based on SNP4232 on chromosome 2. M, DL2000 Plus DNA marker (Vazyme). R and S indicate resistant and susceptible individuals, respectively. Heinong 84 (HN84) and Zhonghuang 13 (ZH13) are also indicated. The PCR amplicons are indicated by solid arrowheads and Hind III-digested fragments are indicated by hollow arrowheads. Statistical analysis was performed using the Fisher’s Exact Test (p = 0.0033).

## Slide 4
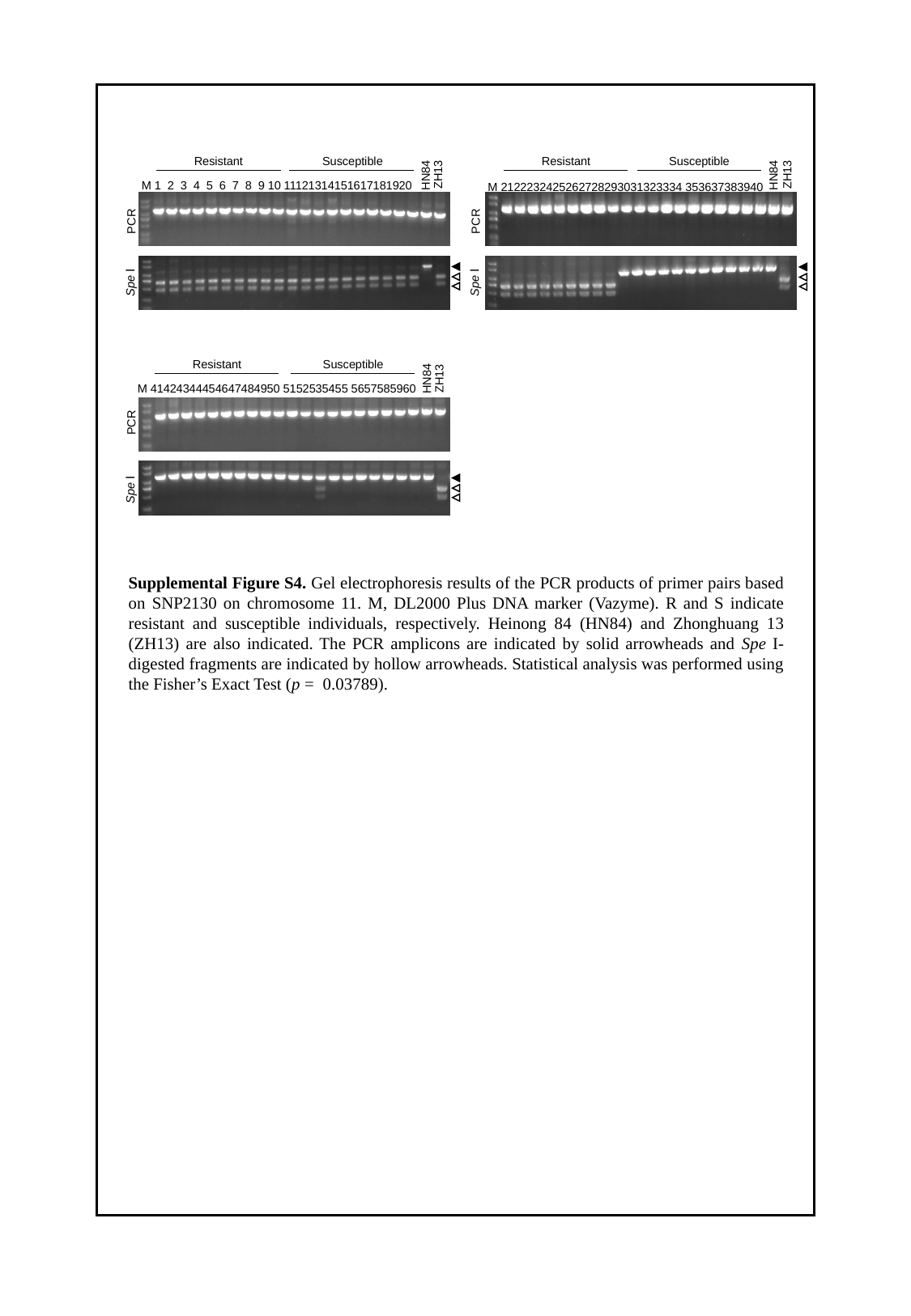

Resistant
Susceptible
Resistant
Susceptible
ZH13
ZH13
HN84
HN84
M 1 2 3 4 5 6 7 8 9 10 11121314151617181920
M 2122232425262728293031323334 353637383940
PCR
PCR
Spe Ⅰ
Spe Ⅰ
Resistant
Susceptible
ZH13
HN84
M 41424344454647484950 5152535455 5657585960
PCR
Spe Ⅰ
Supplemental Figure S4. Gel electrophoresis results of the PCR products of primer pairs based on SNP2130 on chromosome 11. M, DL2000 Plus DNA marker (Vazyme). R and S indicate resistant and susceptible individuals, respectively. Heinong 84 (HN84) and Zhonghuang 13 (ZH13) are also indicated. The PCR amplicons are indicated by solid arrowheads and Spe I-digested fragments are indicated by hollow arrowheads. Statistical analysis was performed using the Fisher’s Exact Test (p = 0.03789).

## Slide 5
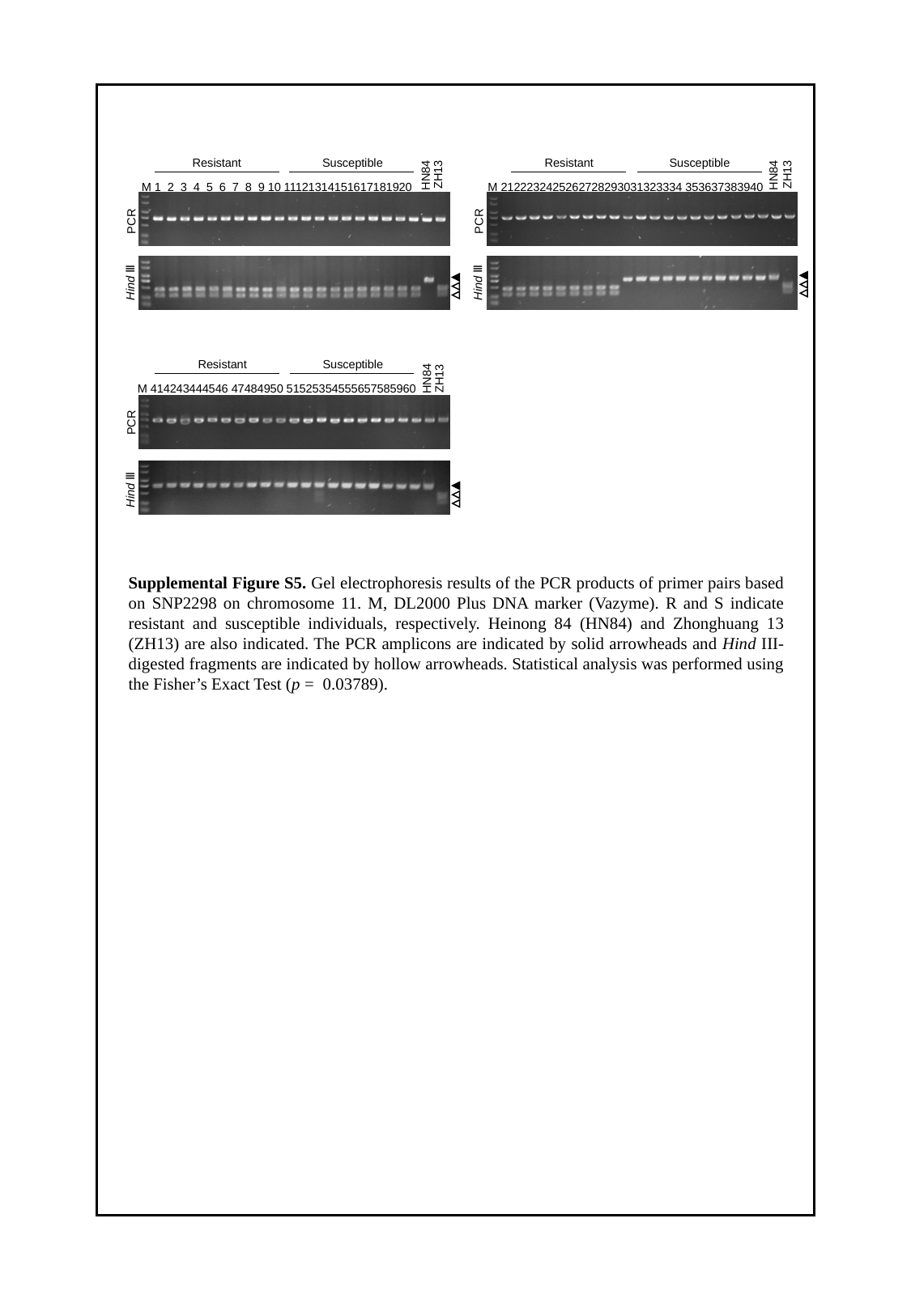

Resistant
Susceptible
Resistant
Susceptible
ZH13
ZH13
HN84
HN84
M 1 2 3 4 5 6 7 8 9 10 11121314151617181920
M 2122232425262728293031323334 353637383940
PCR
PCR
Hind Ⅲ
Hind Ⅲ
Resistant
Susceptible
ZH13
HN84
M 414243444546 47484950 51525354555657585960
PCR
Hind Ⅲ
Supplemental Figure S5. Gel electrophoresis results of the PCR products of primer pairs based on SNP2298 on chromosome 11. M, DL2000 Plus DNA marker (Vazyme). R and S indicate resistant and susceptible individuals, respectively. Heinong 84 (HN84) and Zhonghuang 13 (ZH13) are also indicated. The PCR amplicons are indicated by solid arrowheads and Hind III-digested fragments are indicated by hollow arrowheads. Statistical analysis was performed using the Fisher’s Exact Test (p = 0.03789).
